# P38 A comparative documentary analysis of antifungal policies from NHS trusts participating in BioDrive Antifungal Stewardship

**DOI:** 10.1093/jacamr/dlaf230.045

**Published:** 2025-12-04

**Authors:** Arzoo Dar, Tina Limbu, Florin-Andrei Petra, S Baz, D Allsup, G Barlow, A Hilton

**Affiliations:** Hull York Medical School, UK; Hull York Medical School, UK; Hull York Medical School, UK; Health Sciences, University of York, York, UK; Hull York Medical School, UK; Hull York Medical School, UK; Faculty of Health Sciences, University of Hull, Hull, UK

## Abstract

**Background:**

BioDrive Anti-Fungal Stewardship (AFS) is a National Institute for Health and Care Research (NIHR) funded trial which aims to identify effective ways to detect, prevent and treat fungal infections in haematology patients with acute leukaemia undergoing chemotherapy. These patients are particularly vulnerable to invasive fungal infections due to prolonged immunosuppression. Currently, antifungal medication is often prescribed empirically, which can lead to overtreatment, unnecessary toxicity and growing antimicrobial resistance. Biomarkers such as β-D-glucan and galactomannan can help detect fungal infections before symptom onset, thereby reducing inappropriate antifungal prescribing and enabling more targeted treatment. By supporting timely diagnosis and reduced inappropriate prescribing, this approach directly contributes to antifungal stewardship, a global health priority given the limited availability of antifungal agents. The BioDrive AFS trial has the potential to improve patient outcomes in high-risk haematology patients and inform guidelines on optimal antimicrobial prescribing.

**Objectives:**

This embedded work within the BioDrive trial aimed to: (i) compare antifungal policies for invasive fungal diseases in haematology patients across NHS Trusts; and (ii) identify antifungal blood test biomarkers recommended in policies.

**Methods:**

A documentary analysis was conducted on 17 antifungal policies from NHS Trusts that are sites for, or have had contact with, the BioDrive AFS trial, to compare antifungal stewardship approaches across trusts. To ensure a comprehensive approach, two independent reviewers extracted data using a pre-agreed data extraction matrix, which included criteria such as indications for antifungal therapy, prophylaxis strategies, monitoring protocols and stewardship principles. Disagreements were reviewed and discussed using a structured approach. Extracted data was thematically analysed. Each reviewer generated descriptive comparative reports to highlight common themes, differences and potential areas for improvement in antifungal stewardship practices.

**Results:**

17 policies were analysed. All policies adopted a similar risk stratification system with high-risk patients being identified as primary candidates for antifungal prophylaxis. Most policies 88% (*n*=15/17) recommended mould-active antifungal primary prophylaxis, with posaconazole cited as the first-line prophylactic agent in 80% (*n*=12/15) of policies. Where azoles were contraindicated, AmBisome or caspofungin was recommended in some policies. Eight policies recommended secondary prophylaxis, with voriconazole being the antifungal of choice in 63% (*n*=5/8) of policies. Commonly used biomarkers were galactomannan and β-D-glucan, though data on testing frequency, protocols and turnaround times varied or were unavailable.

**Conclusions:**

Overall, the antifungal prophylaxis policies were similar, particularly regarding risk stratification and first-line prophylaxis choice. However, notable variability was observed in the use of blood test biomarkers and patient monitoring practices, which may translate into differences in clinical care and patient outcomes. These findings highlight areas for further investigation both within the BioDrive AFS trial and across a larger sample of NHS Trusts, to optimize standardized antifungal stewardship practices.

